# Anaemic Streams: Iron and Essential Trace Metals Frequently Limit Primary Producer Biomass

**DOI:** 10.1111/ele.70357

**Published:** 2026-03-08

**Authors:** David M. Costello, Olufemi J. Akinnifesi, Renn C. Schipper, Paisley Kostick, Jordyn T. Stoll, Scott D. Tiegs, Amy M. Marcarelli, Sally A. Entrekin, Raven L. Bier, Krista A. Capps, Dean E. Fletcher

**Affiliations:** ^1^ Department of Biological Sciences Kent State University Kent Ohio USA; ^2^ Department of Biology Grand Valley State University Allendale Michigan USA; ^3^ Department of Biological Sciences Oakland University Rochester Michigan USA; ^4^ Department of Biological Sciences Michigan Technological University Houghton Michigan USA; ^5^ Department of Entomology Virginia Polytechnic Institute Blacksburg Virginia USA; ^6^ Savannah River Ecology Laboratory University of Georgia Aiken South Carolina USA; ^7^ Odum School of Ecology University of Georgia Athens Georgia USA

**Keywords:** biofilm, co‐limitation, metals, nitrogen limitation, phosphorus limitation, zinc

## Abstract

Metals are essential for microbial metabolism, yet their role as limiting nutrients in freshwater streams remains poorly understood. We quantified the prevalence of metal and nutrient (co‐)limitation of primary producers in 41 streams. Metal limitation was widespread with Fe limitation eliciting the strongest and most consistent biomass responses (50% of streams). Zn limitation was also common (33% of streams), marking the first evidence of Zn‐limited stream biofilms at this spatial scale. Metals were often co‐limiting with N and P, highlighting interactions between macro‐ and micronutrients. Diatoms were more responsive to Zn and cyanobacteria reached higher biomass with N and P enrichment, emphasizing divergent nutrient responses among taxa. Predictive modelling indicated that Fe and Zn limitation could be forecasted from environmental variables related to macronutrient supply. These findings challenge the long‐standing assumption that stream primary producers are rarely metal‐limited and suggest that trace metals may play an underappreciated role in regulating stream productivity, community composition and nutrient cycling.

## Graphical abstract

Nutrient enrichment experiments in 41 streams across the eastern United States demonstrate that trace metals can limit the growth of primary producers. Trace metals are frequently co‐limiting with macronutrients and the availability of N and P in streams and watersheds are predictive of Fe and Zn limitation status.

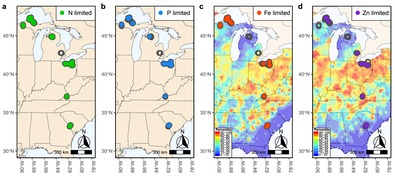

## 1 Introduction

In all ecosystems, the availability of elemental building blocks in the environment is a key factor in determining the taxonomic composition, biomass and diversity of the primary producer community (Falkowski et al. 1998; Lewis and Wurtsbaugh 2008; Stelzer and Lamberti 2001). In recent decades, the classic idea of a single element limiting the growth and biomass of primary producers (i.e., Liebig's Law of the Minimum) has been complemented with a more nuanced understanding of how multiple elements can co‐limit primary producers (Elser et al. 2007; Moore et al. 2013). Co‐limitation can occur at multiple levels of biological organisation; for example, physiological pathways that require multiple elements can cause nutrients to be biochemically linked (Saito et al. 2008), and partitioning of resources among interacting species can allow communities to respond to an increased supply of multiple nutrients (Danger et al. 2008). Advances in understanding limitation and co‐limitation have mostly emerged from study of nitrogen (N) and phosphorus (P); yet more than 20 elements, including metals, are required for life (Kaspari and Powers 2016). Although they are needed in relatively low quantities, metals are essential components of metalloenzymes, which catalyse primary production and nutrient transformations, suggesting metal availability may be limiting or co‐limiting (hereafter (co‐)limiting) factors in certain environments.

Essential metals like iron (Fe), zinc (Zn), molybdenum (Mo) and nickel (Ni) are cofactors for metalloproteins. Metalloproteins are abundant within proteomes of all organisms (Cvetkovic et al. 2010; Mounicou et al. 2009) and catalyse globally important biogeochemical processes such as photosynthesis and N_2_ fixation. In primary producers, Fe supports electron transfer and chemical reduction reactions, most notably photosynthesis, nitrate reduction and N_2_ fixation (Geider and La Roche 1994; Kabata‐Pendias and Szteke 2015; McKay et al. 2001). Similar to Fe, Zn is a component of hundreds of diverse metalloenzymes (Vallee and Auld 1993), including carbonic anhydrase and alkaline phosphatase (Kabata‐Pendias and Szteke 2015; Sunda and Huntsman 1995). Relative to Fe and Zn, Mo and Ni do not support as many functions in metalloenzymes, but they are constituents of the enzymes nitrogenase and nitrate reductase (Mo; Glass et al. 2012; McKay et al. 2001) and urease (Ni; McKay et al. 2001; Rees and Bekheet 1982), which are critical for N acquisition. Because metals are cofactors in enzymes that acquire and transform N and P, metal and nutrient availability may co‐limit primary production (McKay et al. 2001; Saito et al. 2008). For example, limited availability of Mo in the environment can cause organisms to be N limited if N_2_ or nitrate are the only sources of N (Glass et al. 2012); thus, provisioning of Mo or NH_4_^+^ can alleviate limitation. When metals are limiting, the optimal metal at the active site of some enzymes can be replaced by an alternative metal, though the efficiency of the enzyme may be impacted (Saito et al. 2008; Smethurst and Shcherbik 2021). For example, cobalt (Co) and Zn can be co‐limiting because they can be substituted at the active site of carbonic anhydrase, though inorganic carbon uptake is slower with the Co‐bearing enzyme (Morel et al. 1994; Sunda and Huntsman 1995). Although the structure and functional role of metalloenzymes suggests that metal (co‐)limitation is possible in aquatic ecosystems, there is still limited information demonstrating that environmental conditions commonly lead to metal limitation, especially in freshwaters.

The importance of metals as limiting nutrients for primary producers in the ocean has been demonstrated broadly through space and time over the last three decades (Browning and Moore 2023; Coale et al. 1996; Moore et al. 2013). However, evidence for metal limitation of primary production in freshwaters is far more incomplete and dominated by studies in large lakes (e.g., Lewis and Wurtsbaugh 2008; Sterner et al. 2004; Twiss et al. 2005). Certainty about the extent and frequency of metal limitation in the ocean is derived from two lines of evidence: observational studies linking nutrient and metal availability and biological conditions (e.g., Behrenfeld et al. 2009), and organismal response to experimental nutrient and metal enrichment (Moore et al. 2013). In the ocean, dust deposition from terrestrial ecosystems has been linked to alleviation of Fe limitation (Jickells et al. 2005), and extrapolation by researchers has generated the assumption that metal limitation is unlikely to inland waters. However, continental‐scale surveys of rivers and streams in the United States have been used to suggest that Fe influences the diversity and abundance of benthic algae in approximately 50% of the hundreds of streams sampled (Larson et al. 2015; Passy 2010). Although intriguing, these correlative studies have not changed perceptions about the primary limiting nutrients in streams, possibly because of relatively high Fe concentrations in fluvial ecosystems relative to the ocean and a lack of experimental evidence supporting iron limitation. Metal enrichment experiments have been very infrequent in streams, but studies in Costa Rica (Pringle et al. 1986) and the Great Lakes region of the US (Fitzgibbon and Costello 2023) demonstrate that primary producer biomass can be stimulated by metal enrichment. We also know that across aquatic ecosystems, metals can influence microbial community composition (Dengg et al. 2022; Larson et al. 2024; Sorichetti et al. 2016), but there has been little effort to assess whether stream primary producer taxonomic composition is influenced by metal supply. Therefore, conducting metal enrichment experiments across a wider diversity of streams would improve our understanding of the spatial extent of metal limitation in freshwater ecosystems.

**FIGURE 1:**
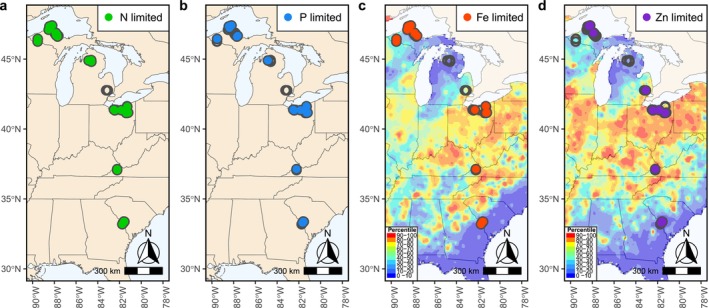
Map of 41 study streams and locations where four of the nutrients (N, P, Fe and Zn) were either limiting or co‐limiting as determined by nutrient diffusing substrates (NDS). Circles are streams where NDS experiments were completed, and filled circles are streams with identified nutrient (co‐)limitation. The background heatmaps in panels c and d show soil A‐horizon Fe and Zn concentration percentiles, respectively (Smith et al. 2014).

In this study, we aimed to expand our understanding of trace metal limitation in freshwaters by conducting nutrient and metal enrichment experiments in 52 streams. These 52 streams spanned 14° latitude across the eastern US (Figure 1), encompassing wide gradients in climate, land cover, soil chemistry, and natural soil metal concentrations (Smith et al. 2014). We selected this spatial gradient to study metal limitation across a wide range of expected metal availability in stream water and primary producer community composition. We designed single and combined nutrient and metal treatments to test for co‐limitation, which was expected based on the functional roles of Fe, Zn, Mo, Ni and Co. In each stream, we measured community composition and biomass responses to nutrient and metal treatments, and we subsequently characterised sites by (co‐)limitation type (Harpole et al. 2011) for nutrients and micronutrients. Consistent with the observational study by Larson et al. (2015), we hypothesized that 50%–75% of streams would be limited or co‐limited by metals, with Fe limitation most common due to the relatively high demand for Fe in algal metabolism. We hypothesized that metals would be most frequently co‐limiting with macronutrients, reflecting established connections between macro‐ and micronutrients in algal physiology (McKay et al. 2001) and similar observations for marine phytoplankton (Browning and Moore 2023). Finally, we hypothesized that watershed characteristics would effectively predict the occurrence of metal limitation across our study streams because of the important role of soils, land use, and upstream features as sources and processors of metals (Larson et al. 2015).

## 2 Methods

### 2.1 Study Sites

Nutrient diffusing substrata (NDS) were placed in 52 streams between the months of June and September in 2021 and 2022 (Figure 1) to target sites when water temperatures and light availability were expected to be sufficiently high to support maximal algal growth, thereby minimising the likelihood that physical constraints would override patterns of limitation (Reisinger et al. 2016; Hauptmann and Myrstener 2023). Storm events, vandalism, and burial compromised experimental results from 11 streams, and here we present results from 41 streams (27 in 2021 and 14 in 2022) where we recovered at least 70% of NDS and at least two NDS from each treatment (Table S1). All regions had at least two streams with successfully recovered NDS, but the distribution of streams was not equal among regions (Table S2).

### 2.2 Nutrient‐Diffusing Substrata (NDS)

NDS were constructed following previously published methods (Fitzgibbon and Costello 2023; Tank et al. 2017) and construction is detailed in the Supporting Information; Methods. We tested 11 treatment combinations of N, P, and metals alone and in combination to assess limitation and co‐limitation. Single nutrient NDS were constructed for N, P, Fe and Zn and these nutrients were combined with themselves (N + P, N + Fe, P + Fe, N + P + Fe, N + Fe + Zn) and with other metals (P + Fe + Ni, P + Fe + Mo) based on biochemical mechanisms of limitation (McKay et al. 2001; Saito et al. 2008). Control NDS with no nutrient addition were included in all experiments. There were slight differences between deployments in 2021 and 2022; in 2021 we included Co with the Zn treatment due to these metals' similar role in carbonic anhydrase and the N + Fe treatment was only used in 2022. All metals and nutrients were added in their most bioavailable form at concentrations intended to alleviate limitation without causing toxicity (Table 1).

**TABLE 1:** Nutrients used in nutrient diffusing substrata (NDS) treatments for assessing nutrient and metal limitation and co‐limitation in study streams. Target doses replicated the mixed‐algal COMBO media (Kilham et al. 1998) and were converted to a cup concentration using formulae in Costello et al. (2016).

Nutrient added	Chemical form	Target dose (μM)	Cup concentration (mM)^a^
N	NH_4_Cl	1000	250
P	KH_2_PO_4_	50	8.9
Fe	FeCl_3_	3.7	0.06
Co^b^	CoCl_2_	0.05	0.011
Zn	ZnSO_4_	0.08	0.0007
Mo	Na_2_MoO_4_	0.09	0.006
Ni	NiCl_2_	0.09	0.015

^a^ Cup concentration varied slightly (±8%) dependent on temperature and length of deployment. Concentrations provided are for 18°C and 21‐day deployment.

^b^ Co was used for 2021 NDS experiments but not 2022.

NDS were deployed in streams by first attaching 1 NDS of each treatment to a PVC L‐bar using zip ties. 5 L‐bars loaded with cups were secured to a concrete block with screws and placed on the bottom of the streambed (Figure S1). Stream sediment was excavated to allow concrete blocks to sit level and flush with the surface of the streambed. The study streams varied in size and water depth, but NDS were deployed in the thalweg at mid‐channel and the overlying water depth was 10–35 cm. NDS were incubated in the stream for 19–29 days (longer for colder streams) before sampling.

### 2.3 NDS Sampling

At sampling, NDS were first moved to a shallow part of the stream and covered with opaque plastic for 20 min prior to field fluorometry to reduce influence of nonphotochemical quenching on fluorescence (Kaylor et al. 2018). NDS were carefully opened and the fritted glass disk was removed and gently rinsed with stream water to remove deposited fine particles. We then measured the concentration of photosynthetic pigments on each cup using a BenthoTorch field fluorometer (bbe moldaenke). The BenthoTorch provided estimates of photosynthetic biomass separated into three groups: cyanobacteria, diatoms and chlorophytes, using the built‐in algorithm. Disks were then frozen for analysis of chlorophyll *a*. In 2021, a single disk from each treatment was preserved in glutaraldehyde for microscopic identification of periphyton and validation of the BenthoTorch. In one stream in 2022 (Manistee River, MI), disks were lost during transport, and we report results from field fluorometry but not lab‐measured chlorophyll *a*.

### 2.4 Water Quality

When NDS were deployed and sampled, filtered (0.7 polyether sulfone) and unfiltered water samples were collected, transported on ice, and stored frozen. A handheld meter was used in the field to measure specific conductivity, pH, and temperature. Major forms of nitrogen and phosphorus were measured in filtered (NO_3_^−^‐N, NH_4_^+^‐N, dissolved reactive phosphorus [DRP], total dissolved N [TDN] and total dissolved P [TDP]) and unfiltered samples (total nitrogen [TN] and total phosphorus [TP]) using a Skalar SAN++ continuous flow analyser. Unfiltered samples were analysed for alkalinity by auto‐titration using a TitraLab AT1000 automatic titrator. Total metals (Mg, Ca, Fe, V, Mn Co, Ni, Cu, Zn, Mo, Cd and Se) were analysed via inductively coupled plasma mass spectrometry (ICP‐MS) on unfiltered water samples (filtered samples were lost during shipment) and detection limits are reported in the Supporting Information; Methods (Table S3).

### 2.5 Statistical Analysis

To evaluate nutrient limitation status in our study streams we used an effect‐size approach as detailed by Harpole et al. (2011). Briefly, we calculated log response ratios for all treatments and interaction response ratios for nutrient combinations to evaluate whether biomass in multi‐element treatments were non‐additive with respect to biomass in single element treatments. For the three‐element treatments, we calculated interaction response ratios by comparing the three‐element biomass to an additive combination of an appropriate two‐element combination to a single metal (e.g., N + Fe + Zn compared to additive N + Fe and Zn). All treatment contrasts for assessing interactions are detailed in the Supporting Information (Table S4). Response ratios were then used to classify each stream by nutrient (co‐)limitation status; log response ratios > 0.326 indicated stimulation of growth and interaction response ratios > 0.385 indicated significant positive non‐additive growth (Harpole et al. 2011). All possible classifications into different forms of limitation and co‐limitation are described in the Supporting Information; Methods (Figure S2). Independent and simultaneous responses to multiple elements were classified as co‐limitation, but we did not consider serial limitation as a form of co‐limitation because it follows the expectations of Liebig limitation (Harpole et al. 2011). We calculated nutrient limitation separately for our lab measured biomass (chlorophyll *a*) and field fluorometry (diatom, cyanobacteria and chlorophyte pigments).

We used random forest predictive models (randomForest package in R; Liaw and Wiener 2002) to evaluate whether environmental conditions were predictive of nutrient limitation status. Due to our limited sample size (*n* = 41 streams) we simplified our response variables to just presence/absence of limitation (primary or co‐limitation) based on the chlorophyll *a* biomass response. For the one stream (Manistee River, MI) where chlorophyll *a* samples were lost, we used the field fluorometer data to classify nutrient limitation. Predictor variables included 27 water quality analytes measured during the experiment, 52 landscape variables describing upstream watersheds (Hill et al. 2016), and 21 variables describing soil metal concentration (as deciles) in watersheds (Smith et al. 2014) (Table S5). Missing water quality data were imputed using predictive mean matching (mice package in R; van Buuren and Groothuis‐Oudshoorn 2011). Less than 1.5% of the data were imputed and at most six observations were imputed for any single variable. Models were trained on data from 80% of the streams and parameters were optimised with a grid search of three tuning parameters: number of variables tested at each split, maximum number of nodes, and number of trees (Table S6). To assess model performance, we report the misclassification rate for the training data and the cross‐validation accuracy for the test data (20% of streams). We considered models to be well‐performing if the misclassification rate for the training data was < 30% and the cross‐validation accuracy for the test data was greater than the random chance ‘no‐information rate’. For well‐performing models, we report partial dependency plots for top predictors.

## 3 Results

**FIGURE 2:**
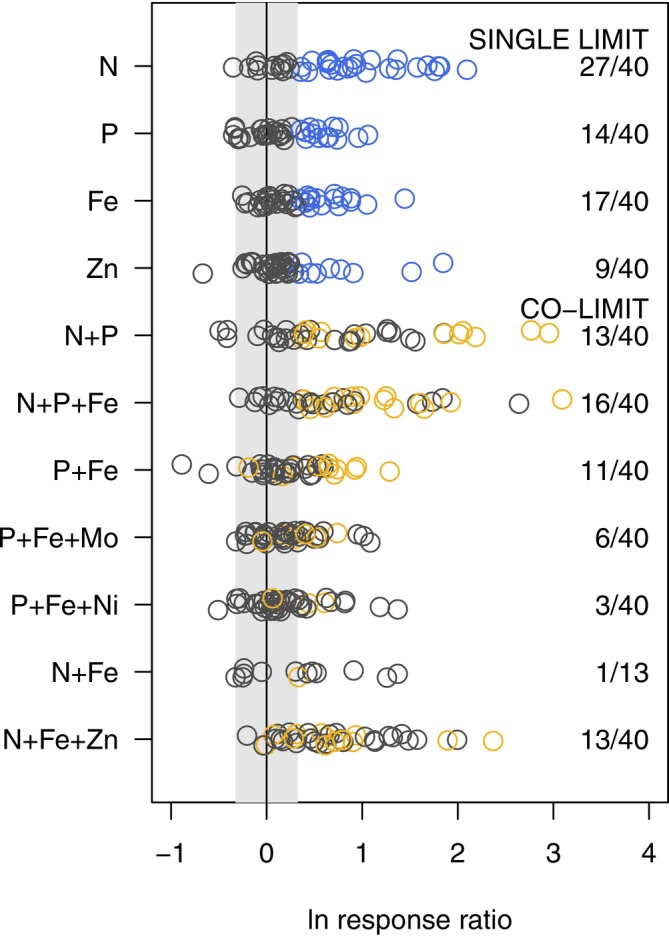
Biofilm chlorophyll *a* response expressed as natural log response ratio (relative to control chlorophyll *a*) under different nutrient enrichment conditions. Positive response ratios outside the grey box are above the threshold of +38.5%, which is an effect size shown to identify nutrient limitation (Harpole et al. 2011). Blue open circles are streams that show single element limitation and gold open circles are streams that show evidence of nutrient co‐limitation. Co‐limitation is identified by comparing responses to single element and combined element treatments as described in Figure S2.

Biofilms in 83% of the streams exhibited a response in chlorophyll *a* biomass that was reflective of nutrient limitation or co‐limitation. N was most frequently (co‐)limiting (75%, 30 of 40 study streams), and N (co‐)limitation was observed across the latitudinal gradient of study (Figure 1a). Single supply of N increased biomass in 27 streams, while the 3 remaining streams that were classified as N limited were solely due to simultaneous co‐limitation with other elements (Figure 2). In approximately half of the study streams, P (20 of 40 streams) and Fe (21 of 40 streams) were identified as (co‐)limiting nutrients. Similar to N, both P and Fe limitation was observed across the latitudinal study gradient, with the exception of an absence of Fe limitation in the streams within the lower peninsula MI (Figure 1b,c). Single supply of P and Fe resulted in greater biomass in 14 and 17 study streams, respectively, with the remainder of P‐ and Fe‐limited streams classified as co‐limited (Figure 2). Single supply of Zn produced greater biomass in nine streams, with four additional streams co‐limited by Zn (33% of streams were Zn limited) (Figure 2). Zn (co‐)limitation was not spatially clustered, but it occurred in all of our study regions (Figure 1d). Mo and Ni were not supplied singly but were identified as co‐limiting with P and Fe in six and three streams, respectively (Figure 2). N‐P‐Fe co‐limitation was the most frequently observed combination of co‐limited elements (16 streams, Figure 2), with N‐P and N‐Fe‐Zn co‐limiting in 13 streams each. Independent co‐limitation was the most common form of co‐limitation among all element combinations; the N + P combination most commonly caused a super‐additive response, whereas multi‐element combinations with metals most commonly caused sub‐additive responses (Table S7).

**FIGURE 3:**
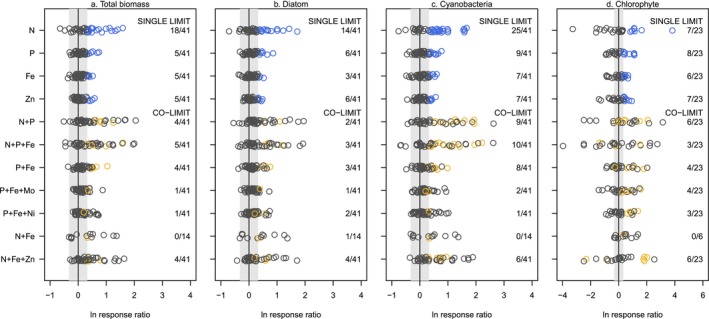
Biofilm biomass response of different primary producer phyla expressed as natural log response ratio (relative to control biomass) under different nutrient enrichment conditions. Positive response ratios outside the grey box are above the threshold of +38.5%, which is an effect size shown to identify nutrient limitation (Harpole et al. 2011). Blue open circles are streams that show single element limitation and gold open circles are streams that show evidence of nutrient co‐limitation. Co‐limitation is identified by comparing responses to single element and combined element treatments as described in Figure S2. Note the change in *x*‐axis in panel d.

Overall, limitation was observed less frequently via field fluorometry than the in‐lab chlorophyll *a* extractions due to lower sensitivity of field fluorometry at high benthic biomass (Figure S3), which has been noted in other studies (Kaylor et al. 2018; Rosero‐López et al. 2021). Although effect sizes were lower, field fluorometry yielded a relative distribution of limitation types similar to chlorophyll *a* biomass—N (co‐)limitation was most frequent, P, Fe and Zn all exhibited single‐element limitation, and all four elements were co‐limiting when combined (Figure 3a). We observed taxonomic differences in response to nutrient combinations across the three major phyla of primary producers. Diatoms were relatively insensitive to nutrient enrichment with smaller response ratios compared to other taxa and chlorophyll *a* (Figure 3b). Diatoms were primarily characterised as N (co‐)limited (14 of 41 streams, 34%) and co‐limitation occurred infrequently (< 10% of streams). Zn (co‐)limitation by diatoms was observed more frequently than Fe (co‐)limitation (six and three streams, respectively). Cyanobacteria frequently exhibited nutrient co‐limitation by N, P and multiple metals (Figure 3b). N (co‐)limitation of cyanobacteria was observed most frequently (25 streams) but P, Fe and Zn (co‐)limitation were all observed at similar frequency (7–9 streams) (Figure 3b). Chlorophytes were only detectible in 23 of our study streams, but their nutrient limitation characteristics were unique from diatoms and cyanobacteria. N (co‐)limitation was not the dominant condition for chlorophytes and single P, Fe and Zn limitation each occurred at a similar frequency (Figure 3d). Although not the focus of this study, negative responses to N (i.e., lower biomass on +N than controls) were also commonly observed for chlorophytes (Figure 3d). Co‐limitation between metals and macronutrients were also common for chlorophyte biomass.

**FIGURE 4:**
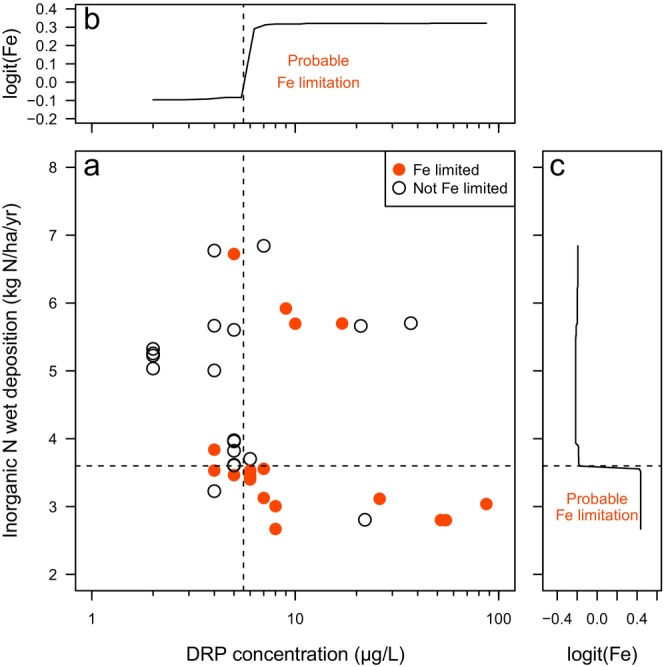
A random forest model indicated that dissolved reactive phosphorus (DRP) concentration and watershed‐scale inorganic nitrogen deposition (N_DEP_) were the best predictors of presence/absence of Fe primary‐ or co‐limitation as identified by nutrient limitation assays. Study streams covered a broad range of DRP and N_DEP_ (a), and Fe (co)limitation (filled orange circles) was most common in streams with low N_DEP_ and high DRP. Partial dependency plots (b, c) from random forest models identified thresholds where the probability of observing Fe limitation increased sharply (DRP > 5.6 μg/L and N_DEP_ < 3.6 kg N/ha/years).

**FIGURE 5:**
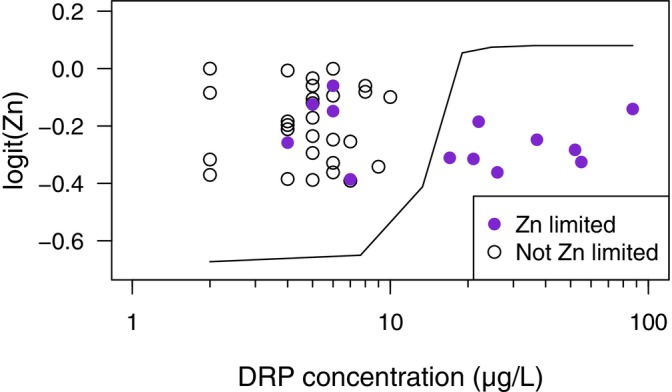
A random forest model indicated that stream dissolved reactive phosphorus (DRP) during the experimental period was the best predictor of presence/absence of Zn primary‐ or co‐limitation as identified by nutrient limitation assays. The partial dependence plot for DRP (solid line) indicates that presence of Zn limitation (filled purple circles) was more likely in streams with DRP > 10 μg/L. Points are jittered in the vertical direction to allow for visualisation of close and overlapping points.

Our random forest models performed better for predicting presence/absence of Fe and Zn (co‐)limitation than for N and P (co‐)limitation (Table S6). The random forest model for N failed to accurately classify any of the streams where N was not limiting in the training dataset, but did accurately predict a single stream in the validation dataset where N was not limiting. Although this model was weak, the top predictors and partial dependency plots were logical with a higher probability of N limitation in small watersheds, with low NO_3_^−^, and less human‐dominated land cover (Figure S4a). The random forest model for P misclassified 45% of the sites in the training dataset and poorly predicted P limitation in the validation dataset (worse than random chance). Overall, this model performed poorly and indicates that the predictor variables we selected cannot accurately predict the presence of P limitation. The random forest model for Fe performed well with moderate accuracy in the training dataset (30% misclassified) and predictions of Fe limitation status for the validation dataset exceeded the no‐information rate (Table S6). Watershed inorganic N wet deposition (N_DEP_) and stream DRP were the strongest predictors of Fe limitation, and stream Mo concentration and watershed area were the next more important variables (Figure S4c). Fe limitation was more probable when N_DEP_ was < 3.6 kg/ha/years and stream DRP concentrations exceeded 5.6 μg/L (Figure 4). 15 of our study streams had DRP and N_DEP_ that met those criteria and all but one demonstrated Fe limitation, and the single stream in that group that was not Fe limited exhibited serial limitation of Fe after N limitation was met. Alternatively, 17 streams had high N_DEP_ and low DRP and only two exhibited Fe limitation. The random forest model for Zn performed well with low misclassification in the training dataset (21%) and high accuracy in the validation dataset (Table S6). Stream DRP was the strongest predictor of Zn limitation with all 8 streams that exceeded 10 μg P/L exhibiting Zn (co‐)limitation (Figure 5). Other variables that were associated with Zn (co‐)limitation were higher soil CaO, low ambient biofilm biomass, and low amounts of anthropogenic N in the watershed (Figure S4d).

## 4 Discussion

Our study revealed that metal limitation of stream primary producers is more common than previously recognised and distributed broadly from the US Great Lakes region to the southeastern US. Because experiments were conducted during a single season, our findings do not capture temporal variation in nutrient or metal limitation, which is well documented in stream ecosystems (e.g., Robinson et al. 2002; Reisinger et al. 2016; Hauptmann and Myrstener 2023). Limitation patterns can shift seasonally as temperature, light, hydrology and nutrient supply change (Beck et al. 2021). Although this is a limitation of our study, our objective was to assess whether trace metal limitation occurs during periods of peak primary productivity, when light and temperature are unlikely to strongly constrain algal growth. Within this context, our results show that metal limitation, particularly by Fe and Zn, can occur during the most biologically productive periods in streams. Fe limitation elicited the strongest and most pervasive response to metal enrichment, which is consistent with observations from marine and lacustrine ecosystems (Browning and Moore 2023; Lewis and Wurtsbaugh 2008). As we hypothesized, approximately 50% of our study streams demonstrated Fe (co‐)limitation, which is aligned with a rate of Fe limitation determined from observational studies of water quality and benthic algae monitoring in US streams (Larson et al. 2015; Passy 2010). This frequency of Fe co‐limitation is greater than the 30% of ocean surface area where phytoplankton are projected to be Fe limited (Behrenfeld et al. 2009). Here, we also provide the first evidence that Zn can limit primary producer biomass in streams. Zn (co‐)limitation was relatively common among streams (33% of streams), but it was not geographically clustered. Zn is recognised as an essential nutrient with broad physiological uses across diverse taxa (Morel et al. 1994; Quigg 2016), but prior to this study little evidence of Zn limitation in freshwater primary producers has been documented. We also observed Ni and Mo co‐limitation, however these elements were limiting much less frequently than the other elements of study.

Overall, the frequency of metal limitation was comparable to macronutrient limitation; Fe‐alone limitation was more common than P‐alone limitation and N‐Fe‐Zn co‐limitation was more common than N‐P co‐limitation. Although the frequency of limitation was similar between metals and macronutrients, metal addition caused a smaller magnitude response in biomass than macronutrients. The median response ratio for N‐limited biofilms was 0.89 (+144%), whereas Fe‐ and Zn‐limited biofilms showed median growth responses of 0.58 (+78%) and 0.66 (+94%), respectively. Additionally, under co‐limitation conditions, fertilisation with N and P most frequently caused super‐additive responses (38% of co‐limited streams), whereas metal‐macronutrient treatments were dominated by sub‐additive growth responses (71% of co‐limited streams). This differential magnitude of response is likely related to the unique physiological roles of these elements. Macronutrients directly contribute to the structural material of biomass, whereas metals indirectly affect growth by altering the efficiency of macronutrient uptake and processing (McKay et al. 2001).

The polyphyletic origins of benthic primary producers have led to divergent nutrient requirements across taxa (Quigg 2016), and thus it is not surprising that we observed differential responses to macronutrient and metal enrichment across our taxonomic divisions. The most apparent patterns in community responses that we observed were diatoms that responded more to Zn than Fe fertilisation, greater cyanobacteria growth under N and P fertilisation than other taxa, and more diverse nutrient requirements for cyanobacteria and chlorophytes. There are no similar studies of freshwater biofilm community response to metals, but comparison to marine and freshwater phytoplankton studies does offer some physiological and genetic support for these observations. Diatom Zn limitation is well established and requirements for Zn have been linked to bicarbonate uptake in marine diatoms (Morel et al. 1994; Saito et al. 2008). The effect of Fe fertilisation on community composition has been equivocal; for example, Marchetti et al. (2012) showed that marine diatoms dominated the phytoplankton community when supplied with Fe, but Dengg et al. (2022) found that cyanobacteria outcompeted diatoms under Fe fertilisation in freshwaters. Our results aligned more with prior freshwater than marine phytoplankton observations, suggesting a potential difference in cyanobacteria and diatom competition between salinity conditions. We also observed that our N and P additions caused disproportionate growth in cyanobacteria and less frequently chlorophyte biomass compared to diatoms. Cyanobacterial dominance under high N has been observed for N‐fixing and non‐diazotrophic cyanobacteria (Chaffin et al. 2013) in lacustrine and marine phytoplankton, although there are exceptions where chlorophytes dominate under high N (Lepori and Robin 2014). Cyanobacteria evolved in the ocean during a time of high metal availability and have a greater physiological demand for metal and a more diverse set of required metals (Merchant and Helmann 2012; Quigg et al. 2003). Although cyanobacterial growth showed a stronger response to a more diverse set of metals than diatoms, the chlorophytes also exhibited similar growth responses to a wide range of metals.

Our predictive models revealed that macronutrient limitation was poorly related to our broad suite of environmental variables, but Fe and Zn limitation could be predicted from environmental variables, in particular those variables that are related to macronutrient supply. Multiple meta‐analyses have demonstrated that macronutrient limitation identified from NDS is related to streamwater inorganic nutrient concentrations, but with a high amount of unexplained variation (Ardón et al. 2021; Beck et al. 2017; Keck and Lepori 2012). Our results concur with those meta‐analyses with inorganic nutrients (i.e., NO_3_ and DRP) among the strongest predictors but overall weak ability to predict sites as N and P limited. Although our landscape and soils data were the most recent data available (from 2019 and 2007–2010, respectively), they were from years prior to our experimental period, and the temporal disconnect may contribute to the poor fit of these models. Our predictive models of Fe and Zn performed well at classifying streams that were metal limited, which suggests that, contrary to N and P, environmental variables can be strong predictors of whether metals are limiting biofilm biomass. Surprisingly, streamwater Fe and Zn concentrations were not among the top predictors, which may reflect the geochemistry of these metals where only a fraction of the total metal concentration in water is bioavailable (Saito et al. 2008). Additionally, 50% of our Zn concentrations were below the method detection limit, thus providing only limited data for our predictive models. Dissolved Fe concentrations are strong predictors of Fe limitation in the ocean and large lakes (Browning and Moore 2023; Havens et al. 2012), and more intensive study of metal bioavailability in freshwaters in concert with limitation experiments is needed to evaluate if there is a relationship between metal concentration and limitation in streams.

For both Fe and Zn limitation models, stream DRP concentration had large explanatory power, and Fe and Zn limitation was more likely with increased DRP concentrations. Pringle et al. (1986) also demonstrated that metals were limiting in Costa Rican streams which were naturally high in P. Using the thresholds derived from our random forest models and the ChemLotUS database (Fernandez et al. 2025), we can project that 76% of stream reaches in the continental US exceed the DRP threshold (> 5.6 μg/L) where Fe limitation is more likely and 61% of stream reaches exceed the Zn threshold (DRP > 10 μg/L). This suggests that our selected study streams are not atypical in their DRP concentrations and there is a high potential for Fe and Zn limitation in streams within the continental US. Fe limitation was also related to N availability, as Fe limitation was predicted from the rate of inorganic N deposition in the watershed. Contrary to the positive relationship with P availability, Fe limitation was more common when N deposition was lower (i.e., below 3.6 kg N/ha/yr). This suggests that Fe and N are co‐limiting where lower N availability in the watershed can induce Fe limitation. This relationship is physiologically feasible because Fe is required for N fixation enzymes and nitrogen reductase (Kabata‐Pendias and Szteke 2015; McKay et al. 2001; Merchant and Helmann 2012). Notably, though we had numerous variables describing N availability in our study streams (e.g., streamwater NO_3_^−^, lithological N, TN:TP), N deposition outperformed other variables in our models. N concentration can be highly variable through time (Guo et al. 2019; Kaushal et al. 2014) and Fe limitation status may reflect longer‐term nutrient demand and supply, a characteristic that may not be made evident through grab samples. Our results of predictive modelling combined with our experimental evidence for N‐P‐Fe and N‐Fe‐Zn co‐limitation emphasises the tight linkage between macronutrients and metals for maximising stream primary producer biomass.

Our data provide evidence that metal limitation in streams is underappreciated and may be widespread, with important implications for how water chemistry governs ecosystem processes in lotic systems. Nutrients, together with light, grazing pressure and disturbance are known to be the primary drivers of stream productivity (Allan et al. 2021), which in turn affects consumer biomass, composition and ultimately ecosystem process rates. However, the conventional paradigm for nutrient limitation in primary producers has focused almost exclusively on N and P. For example, 43% of fertilisation studies found that neither N, P, nor their combination were limiting (Francoeur 2001). Our data suggest this figure likely overestimates the number of locations where primary producers are insensitive to nutrient supply because metals have rarely been considered as nutrients. Recent meta‐analyses similarly report many cases in which N and P additions did not stimulate primary producers (Ardón et al. 2021; Beck et al. 2017; Keck and Lepori 2012), raising the possibility that these systems are limited by other essential elements. Although we seek to expand our understanding of limiting metals, we explored a limited suite of the > 20 metals required for life and not all possible combinations of nutrients. Thus, the prevalence and identity of co‐limiting nutrients in streams may expand further with study of more complex element combinations (Browning and Moore 2023). Because accurate assessment of primary productivity and trophic structure is critical for effective management and remediation (Dodds 2006), recognising metals as potential limiting nutrients may improve our ability to address eutrophication. Although this study focused on algal growth, metals also influence other microbial metabolic functions including many processes linked to N and P cycling (Burgin et al. 2011; McKay et al. 2001). Future studies would benefit from addressing how microbial‐driven processes other than growth rate respond to metal supply, with an emphasis on processes that use metalloenzymes. Broadening the suite of nutrients considered beyond N and P will produce a more complete understanding of the drivers of stream ecosystem structure and function.

## 5 Conclusion

The long‐standing assumption is that stream organisms will not be metal limited due to the close contact between biota and a natural source of metals (lithology), high sediment loads, and relatively high metal concentrations compared to oceans and lakes. However, our data contradict that perspective, repeatedly documenting metal limitation across geographically dispersed locations. Notably, metal limitation was observed for each of the major primary producer phyla; however, discrepancies in growth response to different metals suggest variation among taxa in metal requirements, as expected in evolutionarily divergent groups. Primary producers were frequently co‐limited by macronutrients and metals, and metal (co‐)limitation was most common in streams with elevated streamwater P but low anthropogenic N inputs. Metals catalyse microbial assimilation and transformation of N and P, so metal limitation may have wide‐ranging effects on microbially‐mediated nutrient cycles. Although the importance of metals as limiting nutrients has been established in marine ecosystems for decades (Behrenfeld and Kolber 1999; Moore et al. 2013; Morel et al. 1994), we should exercise caution when translating insights from marine phytoplankton ecology to stream biofilms. Metal concentrations and ratios are more variable through time and space in rivers and streams compared to marine environments (Herndon et al. 2025), with unknown consequences for stream biota. For decades, it has been suggested that trace elements are influencing the ecology of streams (Fitzgibbon and Costello 2023; Larson et al. 2015; Pringle et al. 1986), yet there has been little coordinated effort to study these critical elements. Globally, human activities affect global metal cycles (Rauch and Pacyna 2009), and emerging technologies are projected to increase the demand and exploitation of metals in the near future (Elshkaki et al. 2018). Increased metal concentrations within watersheds are appropriately considered an ecotoxicological risk, but our work suggests they may also act as fertilisers, shifting biofilm communities towards cyanobacterial‐dominated systems. It is imperative to understand the ecological consequences of changing metal availability on the landscape to protect our streams and rivers.

## Author Contributions

David M. Costello, Olufemi J. Akinnifesi, Renn C. Schipper, Paisley Kostick, Jordyn T. Stoll, Amy M. Marcarelli, Sally A. Entrekin, Raven L. Bier, Krista A. Capps and Dean E. Fletcher assisted with the experiments, David M. Costello, Scott D. Tiegs, Amy M. Marcarelli, Sally A. Entrekin, Raven L. Bier, Krista A. Capps and Dean E. Fletcher provided critical field logistics, Olufemi J. Akinnifesi, Renn C. Schipper, Paisley Kostick, Jordyn T. Stoll and Dean E. Fletcher completed sample analysis, David M. Costello wrote the first draft, and all authors edited and revised the manuscript.

## Funding

This work was supported by Division of Environmental Biology, 1943182. National Nuclear Security Administration, DE‐EM0005228.

## Supporting information


**Data S1:** ele70357‐sup‐0001‐Supinfo.pdf.

## Data Availability

The data that support the findings of this study are openly available in Zenodo at https://doi.org/10.5281/zenodo.18497695 (Costello et al. 2026).
