# Supplementary material for: Anaemic Streams: Iron and Essential Trace Metals Frequently Limit Primary Producer Biomass
Source: Ecol Lett. 2026 Mar 8;29(3):e70357. doi: 10.1111/ele.70357 (PMC12967749; doi:10.1111/ele.70357)
Supplement: Supplementary file 1 — Data S1: ele70357‐sup‐0001‐Supinfo.pdf. [file ELE-29-0-s001.pdf]

Supplemental material for:

Anemic streams: Iron and essential trace metals frequently limit primary producer biomass

David M. Costello[1,*], Olufemi J. Akinnifesi[1], Renn C. Schipper[1], Paisley Kostick[1], Jordyn T. Stoll[1,2], Scott D. Tiegs[3], Amy M. Marcarelli[4], Sally A. Entrekin[5], Raven L. Bier[6], Krista A. Capps[6,7], and Dean E. Fletcher[6]

1 Department of Biological Sciences, Kent State University, Kent, OH

2 Department of Biology, Grand Valley State University, Allendale, MI

3 Department of Biological Sciences, Oakland University, Rochester, MI

4 Department of Biological Sciences, Michigan Technological University, Houghton, MI

5 Department of Entomology, Virginia Polytechnic Institute, Blacksburg, VA

6 Savannah River Ecology Laboratory, University of Georgia, Aiken, SC

7 Odum School of Ecology, University of Georgia, Athens, GA

* Corresponding author: dcostel3@kent.edu

Supplemental methods

NDS preparation. Nutrient diffusing substrata (NDS) are a relatively simple experimental approach to fertilize nutrients in streams at the patch scale. NDS are used widely for *in situ* nutrient enrichment studies in aquatic ecology and ecotoxicology. Similar to the prior NDS methods (Costello *et al.* 2016; Fitzgibbon & Costello 2023; Tank *et al.* 2017), nutrients are added to dissolved agar and cooled to form nutrient gels. Attachment substrates are then placed atop the gels and the NDS are attached to streambeds for algal biofilm colonization.

For our study, we constructed NDS using a ½ oz PolyCon® plastic cups fitted with a fritted glass disk to deliver nutrients and trace metals to stream biofilms. Holes (2.2 cm diamater) were punched on the lid of the cups using a leather punch and hammer, creating a hole that exposes fritted glass while securing the disk in place. Each cup was labeled with a unique alphanumeric identifier using paint-based permanent markers.

Agar was prepared at 2% w/v concentration with ultrapure water (resistivity >18 MΩ cm). For a standard batch, we weighed 12 g of agarose into a 1000 mL Erlenmeyer flask, added 600 mL Ultrapure water, and heated the flask on a hot plate with a magnetic stir bar and thermometer. The solution was heated to ~95°C and slowly stirred with close monitoring to prevent boil-over. The flask was removed from the heat, and the agar allowed to cool to 60–70°C. We added N and P as salts and metals from concentrated stock solutions (Table 1) and the solutions were stirred for 2 minutes until fully dissolved and mixed. Prelabelled cups were then filled with the agar solution just below the rim. Cups were left to cool overnight at room temperature for agar to set. The next day, fritted glass disks were gently placed and pressed into the center of each agar surface and lids were closed firmly. Cup lids were secured with vinyl tape.

For immediate deployments, completed NDS units were placed on L bars with zip ties (8-inch long, 5 mm wide), and the L bars were secured on concrete pavers (8x16 inch or 16x16 inch) using a concrete screw. NDS to be deployed >24h after construction were stored in sealed gallon-sized ziptop bags at 4°C and used within 14 days. One extra cup of each batch and treatment was prepared to verify concentrations before field deployment.

Classification of (co-)limitation. Each stream was classified as limited or co-limited by the six elements studied (N, P, Fe, Zn, Ni, & Mo) according to the approach described in Harpole et al. (2011). Quantitative assessment of limitation involves comparison of responses to single element treatments (A and B) and combined treatments (A+B) where the response to single element treatments is calculated as a log response ratio (lnRR):

$$lnRR_A = ln(X_A/X_{control}) \qquad \text{Eq. 1}$$

where X_A and $X_{control}$ are the biomass from the enriched and control treatments, respectively. Any lnRR >0.326 indicates stimulation of growth by the treatment and lnRR < -0.326 indicates growth inhibition. The interaction response ratio is calculated as:

$$RR_{AxB} = [(X_{control} + X_{A+B}) – (X_A + X_B)]/X_{control}$$

Where RR_{AxB} is the response ratio for a specified element combination, and X_{A+B} is the biomass from the combined enrichment treatment. Interaction response ratios are not log transformed because co-limitation is demonstrated by non-additive growth in combined treatments. Any $RR_{AxB} > |0.385|$ was considered a non-additive interaction between A and B (Harpole et al.

2011). There were 7 multi-element treatments that were used to classify co-limitation and the specific element combinations used for classification are provided in Table S4.

Classification of limitation and co-limitation was done by comparing the single element $lnRR_A$, $lnRR_B$, and RR_{AxB} for each stream to the schema in Figure S2. For example, a stream with $lnRR_A$ and $lnRR_B$ both >0.326 (upper right quadrant in Figure S2) was independently co-limited by those two elements, but the specific type of independent co-limitation is defined by the value of RR_{AxB}. For each complete element combination, there were 13 possible classification categories, of which only some were considered evidence of nutrient limitation with a subset of those examples of co-limitation. Classification of negative responses and antagonism were rare and either not considered here due to a focus on nutrient-limited growth (negative responses) or characterized as single element limitation. Serial limitation was not considered a type of co-limitation because this response followed Leibig's Law and may be an experimental artefact (Harpole et al. 2011). Simultaneous (i.e., $lnRR_A < 0.326$, $lnRR_B < 0.326$, and $RR_{AxB} > 0.385$) and independent limitation (i.e., $lnRR_A > 0.326$, $lnRR_B > 0.326$, any value for RR_{AxB}) were considered true co-limitation. We did not include Ni- and Mo-alone treatments due to logistical restraints and thus we assuming that the response to Mo and Ni alone was zero and used a simplified classification schema. If the interaction response ratio (e.g., $RR_{NixP+Fe}$) was >0.385, the stream was considered either independently ($lnRR_{P+Fe} > 0.326$) or simultaneously ($lnRR_{P+Fe} < 0.326$) co-limited by the metal.

Table S1. Number of replicates (lab-measured chlorophyll *a*) for each treatment.

**Stream**	**Region**	**Control**	**N**	**P**	**Fe**	**Zn+Co**	**P+Fe**	**P+Fe+Mo**	**P+Fe+Ni**	**N+Fe+Zn**	**N+P**	**N+P+Fe**
Big Garlic	HMC	4	4	4	4	4	4	4	4	4	4	4
Big Pup	HMC	4	4	4	4	4	4	3	4	4	4	4
Cedar	HMC	4	4	4	2	4	4	4	4	4	4	4
Clark	HMC	4	4	3	4	3	4	4	4	3	4	4
Elm	HMC	4	4	4	4	4	4	4	4	4	4	4
Lost	HMC	4	3	4	4	4	4	4	4	4	4	4
Mountain	HMC	4	4	4	3	4	4	4	4	4	4	4
Pine	HMC	4	4	4	4	4	4	4	4	4	4	4
Salmon Trout	HMC	4	4	4	4	4	4	4	3	4	4	4
Yellow Dog	HMC	4	4	4	4	4	4	4	4	4	4	4
Central	Keweenaw	4	4	4	4	4	4	4	4	4	4	4
Cliff	Keweenaw	4	4	4	4	4	4	4	4	4	4	4
Gratiot	Keweenaw	4	4	4	4	4	4	4	4	4	4	4
Hills	Keweenaw	4	4	4	4	3	4	4	4	3	4	4
Pilgrim	Keweenaw	4	4	4	4	4	4	4	4	4	4	4
Trap Rock	Keweenaw	4	4	4	4	4	4	4	4	4	4	4
Cemetery	NEO	6	5	5	4	5	4	5	5	5	5	5
Chagrin	NEO	5	5	6	5	5	4	5	5	4	4	5
Shady	NEO	5	4	5	5	4	5	4	5	5	3	4
Beaver Dam	SRS	4	4	4	4	4	4	4	4	4	4	4
Crouch	SRS	4	4	4	4	4	4	4	4	4	4	4
McQueen	SRS	4	4	4	4	4	4	4	4	4	4	4
TC5	SRS	4	4	4	4	4	4	4	4	4	4	4
U36	SRS	3	4	4	4	4	4	4	4	4	4	4
U8	SRS	4	4	4	4	4	4	4	4	4	4	4
Tenderfoot	UNDERC	4	4	3	4	4	4	4	5	4	4	4
Trout Brook	UNDERC	4	4	4	4	3	4	4	4	4	4	4

Table S1. (continued)

Stream	Region	Control	N	P	Fe	Zn+ Co	P+Fe	N+Fe	P+Fe+Mo	P+Fe+Ni	N+Fe+Zn	N+P	N+P+Fe
EB Au Sable	Lower MI	5	5	5	5	5	5		5	5	5	5	5
Manistee	Lower MI	5	5	5	5	5	5		5	5	5	5	5
NB Au Sable	Lower MI	4	6	5	5	5	5		5	5	4	5	5
Paint	Lower MI	5	5	5	5	5	5		5	5	5	5	5
Stony	Lower MI	3	4	3	3	3	3		4	4	1	3	2
Breakneck	NEO	5	4	2	5	5	4		3	5	4	5	5
Fish	NEO	5	5	5	5	5	5		5	5	5	5	5
Mahoning	NEO	5	5	4	5	5	5		5	5	5	5	5
Rocky	NEO	5	3	4	4	5	3		5	5	5	5	5
CRO	VA	4	4	5	4	4	3		3	5	3	2	2
FRY	VA	5	5	5	4	5	4		5	5	5	5	5
Chance	Vermillion	4	5	4	4	5	5		4	4	5	5	5
Chapel	Vermillion	5	5	4	5	5	5		5	5	5	4	5
Old Woman	Vermillion	5	5	4	5	5	4		5	5	3	5	5

Table S2. Location of study streams and timing of deployment of nutrient diffusing substrates.

Stream	Region	Latitude	Longitude	Deploy date	Sample date
Big Garlic	HMC	46.66364	-87.62589	2021-07-07	2021-08-05
Big Pup	HMC	46.71093	-87.70455	2021-07-07	2021-08-03
Cedar	HMC	46.82903	-87.93999	2021-07-06	2021-08-02
Clark	HMC	46.61234	-87.69917	2021-07-07	2021-08-03
Elm	HMC	46.84219	-87.86507	2021-07-06	2021-08-02
Lost	HMC	46.7423	-87.73277	2021-07-07	2021-08-05
Mountain	HMC	46.87019	-87.89517	2021-07-08	2021-08-04
Pine	HMC	46.88313	-87.86874	2021-07-08	2021-08-04
Salmon Trout	HMC	46.82229	-87.80576	2021-07-08	2021-08-05
Yellow Dog	HMC	46.71358	-87.8416	2021-07-07	2021-08-03
Central	Keweenaw	47.40272	-88.19727	2021-07-15	2021-08-12
Cliff	Keweenaw	47.373	-88.3117	2021-07-15	2021-08-12
Gratiot	Keweenaw	47.33816	-88.44828	2021-07-15	2021-08-11
Hills	Keweenaw	47.31584	-88.42906	2021-07-15	2021-08-11
Pilgrim	Keweenaw	47.1013	-88.51797	2021-07-15	2021-08-11
Trap Rock	Keweenaw	47.22879	-88.38539	2021-07-15	2021-08-12
Cemetery	NEO	41.60290	-81.28672	2021-09-21	2021-10-14
Chagrin	NEO	41.60063	-81.29075	2021-09-21	2021-10-14
Shady	NEO	41.63211	-81.30451	2021-09-21	2021-10-14
Beaver Dam	SRS	33.1902	-81.7437	2021-10-12	2021-11-03
Crouch	SRS	33.30032	-81.66126	2021-10-11	2021-11-02
McQueen	SRS	33.29683	-81.63076	2021-10-12	2021-11-02
TC5	SRS	33.37337	-81.54988	2021-10-12	2021-11-03
U36	SRS	33.38343	-81.6027	2021-10-12	2021-11-02
U8	SRS	33.29887	-81.67783	2021-10-11	2021-11-02
Tenderfoot	UNDERC	46.25451	-89.53281	2021-07-12	2021-08-08
Trout Brook	UNDERC	46.44464	-89.52144	2021-07-12	2021-08-07
EB Au Sable	Lower MI	44.81282	-84.58903	2022-07-22	2022-08-10
Manistee	Lower MI	44.90178	-84.84501	2022-07-22	2022-08-10
NB Au Sable	Lower MI	44.93201	-84.60474	2022-07-22	2022-08-10
Paint	Lower MI	42.77036	-83.22628	2022-07-19	2022-08-09
Stony	Lower MI	42.76391	-83.07458	2022-07-19	2022-08-09
Breakneck	NEO	41.14911	-81.28266	2022-06-15	2022-07-05
Fish	NEO	41.14667	-81.39634	2022-06-15	2022-07-05
Mahoning	NEO	41.17274	-81.20298	2022-06-15	2022-07-05
Rocky	NEO	41.35076	-81.84574	2022-06-16	2022-07-08
CRO	VA	37.12955	-82.21788	2022-06-27	2022-07-19
FRY	VA	37.06027	-82.21749	2022-06-27	2022-07-19
Chance	Vermillion	41.36739	-82.30947	2022-06-06	2022-07-07
Chapel	Vermillion	41.33696	-82.43618	2022-06-06	2022-07-07
Old Woman	Vermillion	41.38409	-82.51473	2022-06-06	2022-07-07

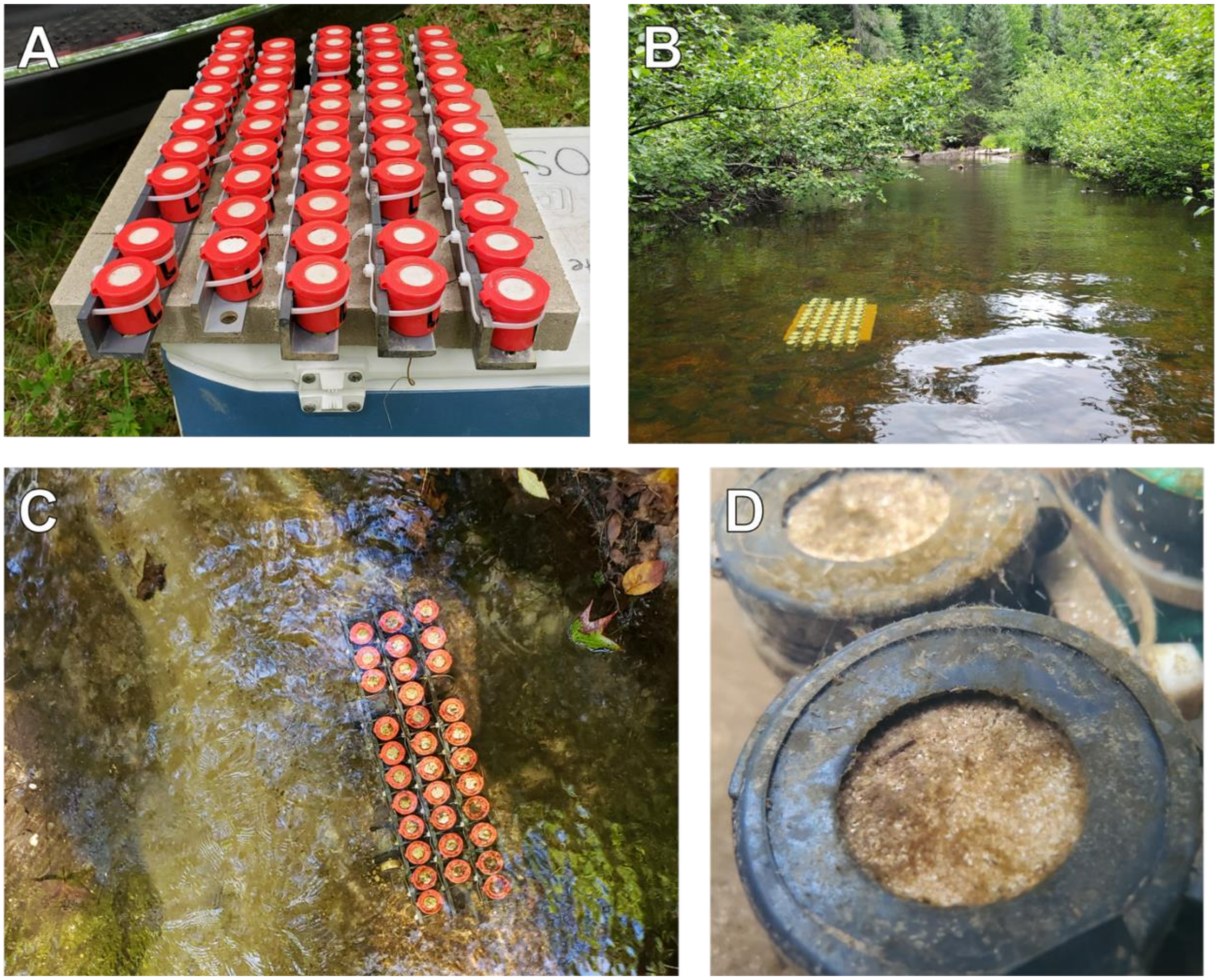

Figure S1. Photos of nutrient diffusing substrate (NDS) used in the study. Prepared cups were attached to concrete pavers (A) and placed flush with the stream bed (B). After 19–29 days, cups were retrieved and algal biomass was measured on the fritted glass disk (C, D).

Table S3. Minimum detection limits for metals analyzed via inductively coupled plasma mass spectrometry.

**Metal**	**Atomic no.**	**Min. detection limit (μg/L)**
Magnesium (Mg)	12	6.3
Calcium (Ca)	20	24
Vanadium (V)	23	0.05
Manganese (Mn)	25	0.06
Iron (Fe)	26	5.2
Cobalt (Co)	27	0.06
Nickel (Ni)	28	0.1
Copper (Cu)	29	0.1
Zinc (Zn)	30	0.6
Selenium (Se)	34	1.1
Molybdenum (Mo)	42	0.15
Cadmium (Cd)	48	0.06

Table S4. Table detailing how interaction effects were calculated to assess co-limitation from 2- or 3-element combination treatments. There were 7 multi-element treatments used a co-limitation was assessed using in formula $[(X_{control} + X_{A+B}) – (X_A + X_B)]/X_{control}$ (Harpole *et al.* 2011). There were no single element treatments of Mo or Ni

**Treatment (A+B)**	**A**	**B**
N+P	N	P
N+P+Fe	N+P	Fe
P+Fe	P	Fe
P+Fe+Mo	P+Fe	control
P+Fe+Ni	P+Fe	control
N+Fe	N	Fe
N+Fe+Zn	N+Fe*	Zn

*Experiments completed in 2021 did not include a N+Fe treatment, so we used an additive model to predict growth in N+Fe from the single N and Fe treatments. In 2022, the N+Fe treatment yielded additive, sub-additive, or antagonistic responses to the combination of N and Fe, thus the additive approach in 2021 is conservative with respect to assessing Zn co-limitation.

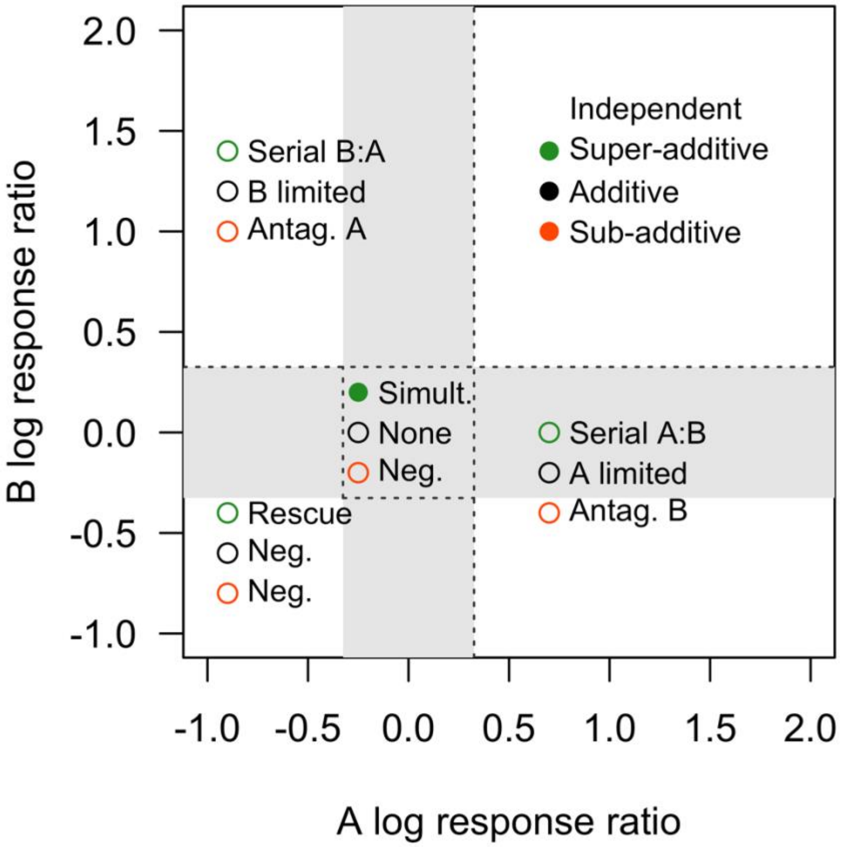

Figure S2. Conceptual model for how biofilm nutrient limitation status was determined from two nutrients (A and B) supplied alone and in combination. We calculated log response ratios of biomass with a nutrient relative to the control, and used the threshold identified by Harpole and colleagues (2011) to signify stimulation (>0.326) or inhibition (< -0.326) of growth. The response ratios when exposed to nutrient A and B alone placed each stream into one of 5 regions on the biplot (separated by the dotted lines) used for classification. The primary producer biomass when exposed to the combined nutrient treatments (A+B) was used to calculate an interaction response ratio (see Harpole *et al.* 2011 Eq. 5 and Fig. 1), which allowed for classification into the sub-categories in green, black, and red in each region (green: >0.385, black: -0.385–0.385, red: < -0.385). All classifications that are identified with a filled symbol are different types of true nutrient co-limitation. Antag. = Antagonistic, Simult. = Simultaneously co-limited; Neg. = Negative response.

Table S5. Predictor variables used in all random forest models. Landscape variables are summed or averaged over the entire upstream watershed area.

Variable code	Full name	Units	Data source
Control	Control NDS chlorophyll *a*	ug/cm^2	This study
WSAREASQKM	Watershed area	km^2	StreamCat (Hill *et al.* 2016)
SANDWS	Soil sand content	%	StreamCat
CLAYWS	Soil clay content	%	StreamCat
MANUREWS	Mean manure application rate	kg/ha/yr	StreamCat
FERTWS	Synthetic nitrogen fertilizer application mean rate	kg/ha/yr	StreamCat
CBNFWS	Cultivated biological nitrogen fixation mean rate	kg/ha/yr	StreamCat
CHEMWS	Water chemistry index score	Unitless	StreamCat
BFIWS	Base flow index	%	StreamCat
NABD_DENSWS	NABD dam density	count/km^2	StreamCat
RUNOFFWS	Mean runoff	mm	StreamCat
NPDESDENSWS	NPDES site density	count/km^2	StreamCat
SUPERFUNDDENSWS	Superfund site density	count/km^2	StreamCat
TRIDENSWS	Density of toxic release inventory sites	count/km^2	StreamCat
PRECIP8110WS	30-year mean annual precipitation	mm	StreamCat
TMEAN8110WS	30-year mean annual air temperature	°Celsius	StreamCat
WWTPALLDENSWS	All wastewater treatment density	count/km^2	StreamCat
SW_FLUXWS	Surface water nitrogen flux	kg N/km^2	StreamCat
INORGNWETDEP2008WS	Precipitation-weighted mean annual wet deposition of NH_3+NO_3	kg/ha/yr	StreamCat
SN2008WS	Annual mean sulfur and nitrogen wet deposition	kg/ha/yr	StreamCat
NWS	Mean lithological nitrogen	%	StreamCat
PCTDECID2019WS	Deciduous forest area	%	StreamCat
PCTCONIF2019WS	Evergreen forest area	%	StreamCat
PCTURBHI2019WS	Developed, high intensity land use area	%	StreamCat
PCTURBMD2019WS	Developed, medium intensity land use area	%	StreamCat
PCTURBLO2019WS	Developed, low intensity land use area	%	StreamCat
PCTURBOP2019WS	Developed, open space land use area	%	StreamCat
PCTCROP2019WS	Row crop area	%	StreamCat
PCTHAY2019WS	Pasture/hay area	%	StreamCat

PCTMXFST2019WS	Mixed deciduous/evergreen forest area	%	StreamCat
MGOWS	Mean lithological magnesium oxide in surface soil	weight %	StreamCat
K2OWS	Mean lithological potassium oxide in surface soil	weight %	StreamCat
AL2O3WS	Mean lithological aluminum oxide in surface soil	weight %	StreamCat
NA2OWS	Mean lithological sodium oxide in surface soil	weight %	StreamCat
SIO2WS	Mean lithological silicon dioxide in surface soil	weight %	StreamCat
CAOWS	Mean lithological calcium oxide in surface soil	weight %	StreamCat
P2O5WS	Mean lithological phosphorous oxide in surface soil	weight %	StreamCat
SWS	Mean lithological sulfur in surface soil	weight %	StreamCat
FE2O3WS	Mean lithological ferric oxide in surface soil	weight %	StreamCat
PERMWS	Mean permeability	%	StreamCat
RCKDEPWS	Mean bedrock depth	cm	StreamCat
OMWS	Mean organic matter content in surface soil	%	StreamCat
MINEDENSWS	Mine density	count/km^2	StreamCat
SEPTICWS	Septic system density	kg/ha/yr	StreamCat
NANIWS	Net anthropogenic nitrogen	kg/ha/yr	StreamCat
NSURPWS	Nitrogen surplus	%	StreamCat
RDDENSWS	Mean road density	%	StreamCat
COALMINEDENSWS	Coal mine density	count/km^2	StreamCat
PCTIMP2019WS	Mean imperviousness	%	StreamCat
PTSOURCE	Point source density – Sum of coal mine, mine, waste water treatment plant, NPDES site, superfund site, and toxic release inventory site density	count/km^2	StreamCat
PCTURB	Sum of high intensity, medium intensity, low intensity, and open space land use	%	StreamCat
PCTFOR	Total forest cover, combining mixed forest cover, evergreen cover, and deciduous cover	%	StreamCat
PCTAG	Total agriculture land cover, sum of crop cover and hay/pasture cover	%	StreamCat
A_Co	Cobalt concentration in the A horizon	mg/kg	Smith et al. 2014

A_Cu	Copper concentration in the A horizon	mg/kg	Smith et al. 2014
A_Fe	Iron concentration in the A horizon	weight %	Smith et al. 2014
A_Mo	Molybdenum concentration in the A horizon	mg/kg	Smith et al. 2014
A_Ni	Nickel concentration in the A horizon	mg/kg	Smith et al. 2014
A_P	Phosphorus concentration in the A horizon	mg/kg	Smith et al. 2014
A_Zn	Zinc concentration in the A horizon	mg/kg	Smith et al. 2014
C_Co	Cobalt concentration in the C horizon	mg/kg	Smith et al. 2014
C_Cu	Copper concentration in the C horizon	mg/kg	Smith et al. 2014
C_Fe	Iron concentration in the C horizon	weight %	Smith et al. 2014
C_Mo	Molybdenum concentration in the C horizon	mg/kg	Smith et al. 2014
C_Ni	Nickel concentration in the C horizon	mg/kg	Smith et al. 2014
C_P	Phosphorus concentration in the C horizon	mg/kg	Smith et al. 2014
C_Zn	Zinc concentration in the C horizon	mg/kg	Smith et al. 2014
Top5_Co	Cobalt concentration in soil top 5 cm	mg/kg	Smith et al. 2014
Top5_Cu	Copper concentration in soil top 5 cm	mg/kg	Smith et al. 2014
Top5_Fe	Iron concentration in soil top 5 cm	weight %	Smith et al. 2014
Top5_Mo	Molybdenum concentration in soil top 5 cm	mg/kg	Smith et al. 2014
Top5_Ni	Nickel concentration in soil top 5 cm	mg/kg	Smith et al. 2014
Top5_P	Phosphorus concentration in soil top 5 cm	mg/kg	Smith et al. 2014
Top5_Zn	Zinc concentration in soil top 5 cm	mg/kg	Smith et al. 2014
Mg_ugL	Magnesium concentration in the water column	µg/L	This study
Ca_ugL	Calcium concentration in the water column	µg/L	This study
Fe_ugL	Iron concentration in the water column	µg/L	This study
V_ugL	Vanadium concentration in the water column	µg/L	This study
Mn_ugL	Manganese concentration in the water column	µg/L	This study
Co_ugL	Cobalt concentration in the water column	µg/L	This study
Ni_ugL	Nickel concentration in the water column	µg/L	This study

Cu_ugL	Copper concentration in the water column	µg/L	This study
Zn_ugL	Zinc concentration in the water column	µg/L	This study
Mo_ugL	Molybdenum concentration in the water column	µg/L	This study
Cd_ugL	Cadmium concentration in the water column	µg/L	This study
Se_ugL	Selenium concentration in the water column	µg/L	This study
NO3_ugL	Nitrate concentration in the water column	µg/L	This study
SRP_ugL	Dissolved reactive phosphorus concentration in the water column	µg/L	This study
NH4_ugL	Ammonium concentration in the water column	µg/L	This study
TDN_ugL	Total dissolved nitrogen concentration in the water column	µg/L	This study
TDP_ugL	Total dissolved phosphorus concentration in the water column	µg/L	This study
TN_ugL	Total nitrogen concentration in the water column	µg/L	This study
TP_ugL	Total phosphorus concentration in the water column	µg/L	This study
Alk_mgL	Water column alkalinity	mg/L	This study
pH	Water column pH	pH	This study
Cond_uScm	Water column conductivity	uS/cm	This study
TNTP	Molar ratios of water column TN and TP concentrations	Molar ratio	This study
NO3SRP	Molar ratios of water column NO_3 and DRP concentrations	Molar ratio	This study
TNFe	Molar ratios of water column TN and Fe concentrations	Molar ratio	This study
TPFe	Molar ratios of water column TP and Fe concentrations	Molar ratio	This study

Table S6. Tuning parameters and model fits for random forest models predicting nutrient limitation of biofilm biomass from environmental parameters. Response variables were presence/absence of limitation (primary or co-limitation) in the study stream as determined by chlorophyll *a* biomass on NDS. Potential predictor variables included 27 point-measurements of water quality during the experiment, 52 landscape variables describing upstream watersheds, and 21 variables describing soil metal concentrations in watersheds. The 41 streams were split into 80% for training the model and 20% for testing. Models with misclassification rates ≤33% (training data) and cross-validation accuracy (test data) greater than the no-information rate (NIR) were considered well-performing models.

**Nutrient**	**Variables at split**	**Maximum nodes**	**Number of trees**	**Misclass. rate (Yes; No)**[a]	**Cross-val. Accuracy/NIR**[b]
Nitrogen	10	5	1100	2/25; 8/8	0.75/0.75
Phosphorus	45	3	1500	7/16; 8/17	0.50/0.63
Iron	90	4	700	5/17; 5/16	0.75/0.50
Zinc	65	5	600	4/10; 3/23	0.88/0.63

a. Misclassification rate is for the train data only (n = 33); b. cross validation accuracy of the test data (n = 8).

Table S7. Types of co-limitation observed under different element combinations with biofilm chlorophyll *a* as a response variable. The classification schema for co-limitation is detailed in Figure S2 and Table S2. 40 total streams were assessed, with the exception of N-Fe (13 streams).

Co-limit type	N-P	P-Fe	N-Fe	N-P-Fe	N-Fe-Zn
Independent – sub-additive	4	8	1	12	8
Independent – additive	2	2	0	1	1
Independent – super-additive	5	0	0	1	0
Simultaneous	2	1	0	2	4
Total	13	11	1	16	13

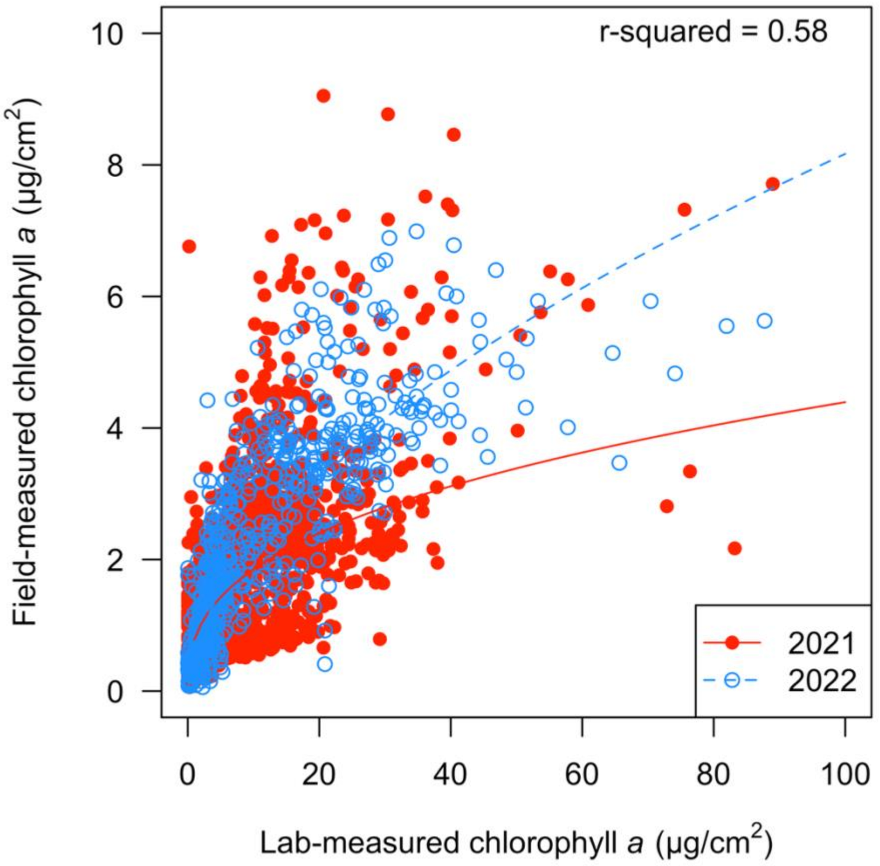

Figure S3. Relationship between chlorophyll *a* measured in the lab via extraction and spectrometry and in the field using a field fluorometer (BenthoTorch). There is a strong relationship between these two measures of chlorophyll *a* but the field fluorometer was saturated at high biomass. Saturation occurred at lower concentrations in 2021 compared to 2022.

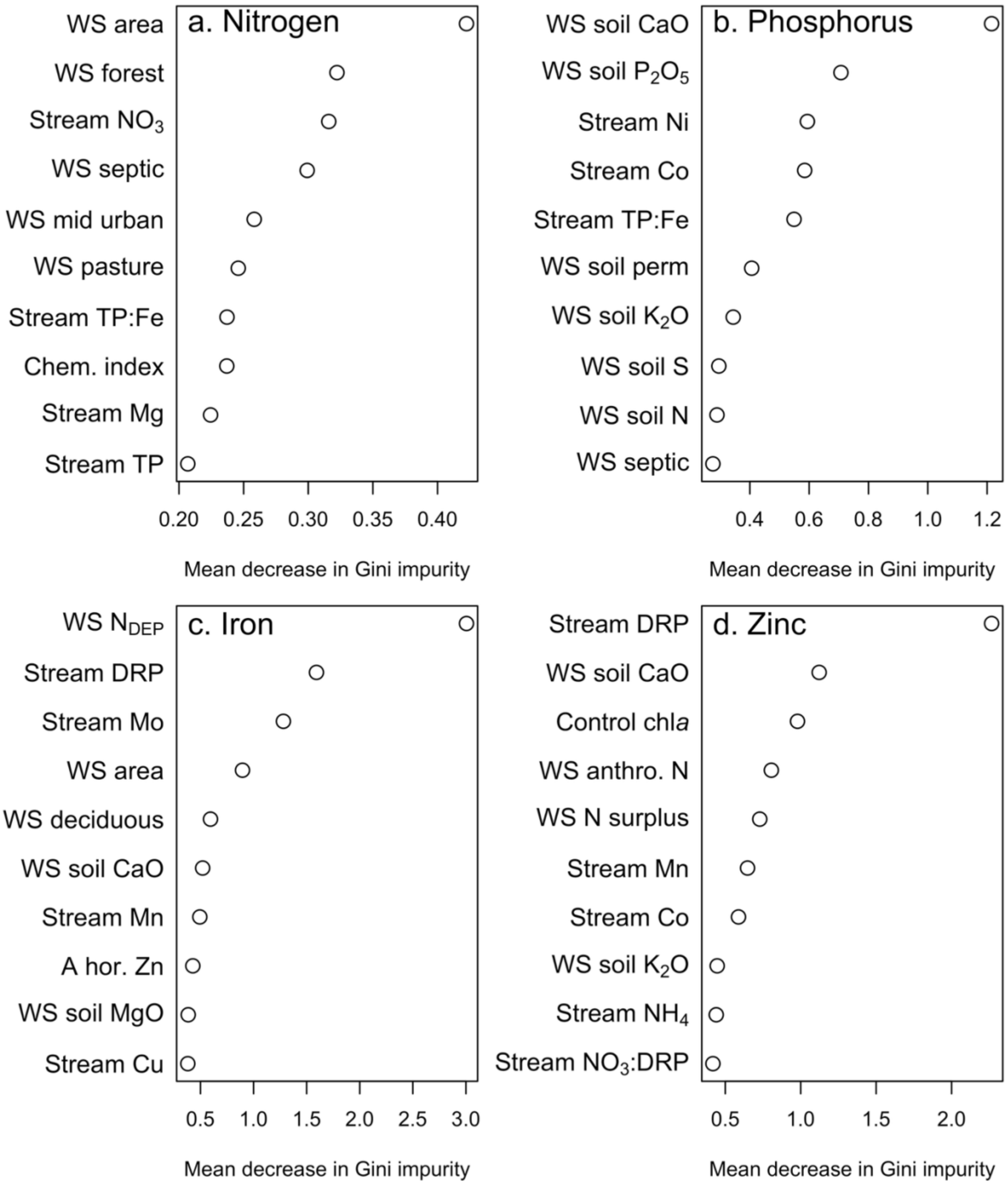

Figure S4. Variable importance for random forest models predicting nutrient limitation (primary or co-limitation) as determined by chlorophyll *a* response on NDS in 41 streams. Each model included 100 potential predictor variables and the top 10 variables, ranked based on mean decrease in Gini impurity, are reported. Models predicting nitrogen (a) and phosphorus (b) limitation were considered poor base on high misclassification rate on the training data (80%) and poor cross-validation accuracy on test data (Table S4).

Supplemental references

Costello, D.M., Rosi-Marshall, E.J., Shaw, L.E., Grace, M.R. & Kelly, J.J. (2016). A novel method to assess effects of chemical stressors on natural biofilm structure and function. *Freshw Biol*, 61, 2129–2140.

Fitzgibbon, A.S. & Costello, D.M. (2023). Trace metal–macronutrient colimitation of algal biofilms in streams with differing ambient inorganic nutrients. *Freshwater Science*, 42, 285–295.

Harpole, W.S., Ngai, J.T., Cleland, E.E., Seabloom, E.W., Borer, E.T., Bracken, M.E.S., *et al.* (2011). Nutrient co-limitation of primary producer communities. *Ecol Lett*, 14, 852–862.

Hill, R.A., Weber, M.H., Leibowitz, S.G., Olsen, A.R. & Thornbrugh, D.J. (2016). The Stream-Catchment (StreamCat) Dataset: A Database of Watershed Metrics for the Conterminous United States. *J Am Water Resour Assoc*, 52, 120–128.

Smith, D.B., Cannon, W.F., Woodruff, L.G., Solano, F. & Ellefsen, K.J. (2014). *Geochemical and mineralogical maps for soils of the conterminous United States*.

Tank, J.L., Reisinger, A.J. & Rosi, E.J. (2017). Nutrient limitation and uptake. In: *Methods in Stream Ecology - Volume 2: Ecosystem Function* (eds. Lamberti, G.A. & Hauer, F.R.). Academic Press, pp. 147–171.
